# A computationally feasible multi-trait single-step genomic prediction model with trait-specific marker weights

**DOI:** 10.1186/s12711-024-00926-2

**Published:** 2024-08-16

**Authors:** Ismo Strandén, Janez Jenko

**Affiliations:** 1 Natural Resources Institute Finland (Luke), Jokioinen, Finland; 2 Geno SA, Storhamargata 44, 2317 Hamar, Norway

## Abstract

**Background:**

Regions of genome-wide marker data may have differing influences on the evaluated traits. This can be reflected in the genomic models by assigning different weights to the markers, which can enhance the accuracy of genomic prediction. However, the standard multi-trait single-step genomic evaluation model can be computationally infeasible when the traits are allowed to have different marker weights.

**Results:**

In this study, we developed and implemented a multi-trait single-step single nucleotide polymorphism best linear unbiased prediction (SNPBLUP) model for large genomic data evaluations that allows for the use of precomputed trait-specific marker weights. The modifications to the standard single-step SNPBLUP model were minor and did not significantly increase the preprocessing workload. The model was tested using simulated data and marker weights precomputed using BayesA. Based on the results, memory requirements and computing time per iteration slightly increased compared to the standard single-step model without weights. Moreover, convergence of the model was slower when using marker weights, which resulted in longer total computing time. The use of marker weights, however, improved prediction accuracy.

**Conclusions:**

We investigated a single-step SNPBLUP model that can be used to accommodate trait-specific marker weights. The marker-weighted single-step model improved prediction accuracy. The approach can be used for large genomic data evaluations using precomputed marker weights.

## Background

Genes influencing traits evaluated in livestock production, such as milk production and fertility, are usually unknown. Therefore, most genomic evaluations in practice use about 50,000 single nucleotide polymorphisms (SNPs) and they are a-priori assumed to have the same weight. However, if the most important SNPs or regions of the genome for a trait are identified and given more weight in the genomic prediction model, the accuracy of the model can be improved [1, 2]. These and other studies have used a single-trait model. In practice, genetic evaluations of dairy cattle often use a multi-trait model, and the most advanced model is the single-step genomic model [3, 4]. A computationally efficient approach for including marker weights in a multi-trait single-step model has not been presented.

Single-trait single-step genomic best linear unbiased prediction (GBLUP) models typically use equal marker weights in the genomic relationship matrix [1, 5] which is inverted and used in standard software to estimate genomic breeding values (GEBV). In this approach, even single-step GBLUP using the algorithm for proven and young [6] and the T relationship matrix [7] (GTBLUP) approaches can be used, provided the preprocessing step to compute the necessary matrices allows the inclusion of weights in the computations. This has been tested to work in single-trait single-step models [1]. The use of marker weights is equivalent to using different variances for the markers.

Brøndum et al. [2] investigated GBLUP types of models and observed that adding quantitative trait loci (QTL) markers improved prediction reliability. Specifically, when the added QTL markers were included using a separate variance component, the model could better emphasize these known QTL. When using the GBLUP model to include QTL markers in the analysis, reliability was increased by up to 4 percentage points for production traits in Nordic Holsteins, up to 3 percentage points for Nordic Red dairy cattle, and up to 5 percentage points for French Holsteins. Zhang et al. [1] presented and investigated several approaches for computing marker weights for single-trait single-step models. They observed that the marker-weighted single-step method gave more accurate GEBV than GBLUP, BayesC, and BayesB. Likewise, Fragomeni et al. [5] observed that adding selected variants slightly increased the reliabilities of single-step GBLUP GEBV for stature in US Holstein.

Genetic evaluation software usually applies the same marker weights to all traits in a multi-trait model. Using different marker weights for each trait requires a different genomic relationship matrix for each trait variance and trait-by-trait covariance, e.g., [8]. This increases computations in the preprocessing and solving steps, compared to using no marker weights and a common genomic relationship matrix for all traits. Single-step SNPBLUP models [9–12] are equivalent to single-step GBLUP, but avoid the use of a genomic relationship matrix by explicitly including marker effects in the model. Modeling marker effects explicitly may allow for a computationally simpler approach for the inclusion of marker-specific weights because they do not need to be included in a genomic relationship matrix and can be incorporated into the marker covariance matrix.

Karaman et al. [13] investigated different ways of assigning weights or variances to SNPs in genomic prediction models. They developed a Bayesian multi-trait regression model (BayesN0) where SNPs are grouped by genomic region which may have different covariance structures. This model was also applied in a single-step framework (ssBayesN0). The ssBayesN0 model gave higher prediction accuracies than the BayesN0 model. Their results suggested that using ssBayesN0 to estimate the marker (co)variance components and then applying them in a multi-trait single-step SNPBLUP approach could be a good strategy for routine genomic evaluations. However, a multi-trait Bayesian model is computationally demanding and not feasible for genetic evaluation of large genomic data sets.

In this study, we present and implement a multi-trait single-step SNPBLUP approach with trait-specific marker weights that can be used to analyze large data sets with many genotyped animals. We consider a two-step approach, where the first step estimates the marker weights and the second step uses them in the single-step model. Weights between trait covariances or marker-specific (co)variance matrices can be used in the model as well. The approach is tested on simulated data by single-step models with or without marker weights. The analyses are contrasted by their computational efficiency and prediction ability.

## Methods

### Multi-trait single-step genomic model

We follow the notation presented in [14] but extend it to a multi-trait model. Consider a multi-trait linear mixed model for single-step GBLUP for *T* traits. The model for trait *i* is:

1 $${\mathbf{y}}_{i}={\mathbf{X}}_{i}{\mathbf{b}}_{i}+\left[\begin{array}{cc}{\mathbf{W}}_{i,n}& \boldsymbol{0}\\ \boldsymbol{0}& {\mathbf{W}}_{i,\text{g}}\end{array}\right]\left[\begin{array}{c}{\mathbf{u}}_{i,n}\\ {\mathbf{u}}_{i,\text{g}}\end{array}\right]+{\mathbf{e}}_{i},$$

where ${\mathbf{y}}_{i}$ is the vector of trait $i$ observations, ${\mathbf{b}}_{i}$ is the vector of trait $i$ fixed effects, ${\mathbf{u}}_{i,n}$ is the vector of trait $i$ additive genetic effects for the non-genotyped animals, ${\mathbf{u}}_{i,\text{g}}$ is the vector of trait $i$ additive genetic effects for the genotyped animals, and ${\mathbf{e}}_{i}$ is the vector of trait $i$ residuals. The matrices ${\mathbf{X}}_{i}$, ${\mathbf{W}}_{i,n}$, and ${\mathbf{W}}_{i,\text{g}}$ relate records in ${\mathbf{y}}_{i}$ to the corresponding effects.

The linear model (1) can be expressed simpler by combining vectors and matrices. Denote $${\mathbf{u}}_{i}=\left[\begin{array}{c}{\mathbf{u}}_{i,n}\\ {\mathbf{u}}_{i,\text{g}}\end{array}\right]$$ and $${\mathbf{W}}_{i}=\left[\begin{array}{cc}{\mathbf{W}}_{i,n}& \boldsymbol{0}\\ \boldsymbol{0}& {\mathbf{W}}_{i,\text{g}}\end{array}\right]$$ for the additive genetic effects of trait $i$. Furthermore, let $$\mathbf{y}=\left[\begin{array}{c}{\mathbf{y}}_{1}\\ \vdots \\ {\mathbf{y}}_{T}\end{array}\right]$$, $$\mathbf{b}=\left[\begin{array}{c}{\mathbf{b}}_{1}\\ \vdots \\ {\mathbf{b}}_{T}\end{array}\right]$$, $$\mathbf{u}=\left[\begin{array}{c}{\mathbf{u}}_{1}\\ \vdots \\ {\mathbf{u}}_{T}\end{array}\right]$$, and $$\mathbf{e}=\left[\begin{array}{c}{\mathbf{e}}_{1}\\ \vdots \\ {\mathbf{e}}_{T}\end{array}\right]$$, and similarly for the matrices $$\mathbf{X}=\left[\begin{array}{ccc}{\mathbf{X}}_{1}& \boldsymbol{0}& \boldsymbol{0}\\ \boldsymbol{0}& \ddots & \boldsymbol{0}\\ \boldsymbol{0}& \boldsymbol{0}& {\mathbf{X}}_{T}\end{array}\right]$$ and $$\mathbf{W}=\left[\begin{array}{ccc}{\mathbf{W}}_{1}& \boldsymbol{0}& \boldsymbol{0}\\ \boldsymbol{0}& \ddots & \boldsymbol{0}\\ \boldsymbol{0}& \boldsymbol{0}& {\mathbf{W}}_{T}\end{array}\right]$$. Then, the model can be written as:

2 $$\mathbf{y}=\mathbf{X}\mathbf{b}+\mathbf{W}\mathbf{u}+\mathbf{e}.$$

The assumed distribution for the additive genetic effect in the models (1) and (2) is $\mathbf{u}\sim MVN\left(\boldsymbol{0},{\mathbf{G}}_{0}\otimes \mathbf{H}\right)$, where ${\mathbf{G}}_{0}$ is the variance–covariance matrix for the additive effects and $\mathbf{H}$ combines pedigree and genomic relationship among animals. Assume the residual covariance matrix to be $\mathbf{R}$, i.e., $\mathbf{e}\sim MVN\left(\boldsymbol{0},\mathbf{R}\right)$.

#### Marker weights in single-step GBLUP

The mixed model equations (MME) for the multi-trait model (2) are:

3 $$\left[\begin{array}{cc}{\mathbf{X}}^{\mathbf{^{\prime}}}{\mathbf{R}}^{-1}\mathbf{X}& {\mathbf{X}}^{\mathbf{^{\prime}}}{\mathbf{R}}^{-1}\mathbf{W}\\ {\mathbf{W}}^{\mathbf{^{\prime}}}{\mathbf{R}}^{-1}\mathbf{X}& {\mathbf{W}}^{\mathbf{^{\prime}}}{\mathbf{R}}^{-1}\mathbf{W}+{\mathbf{G}}_{0}^{-1}\otimes {\mathbf{H}}^{-1}\end{array}\right]\left[\begin{array}{c}\widehat{\mathbf{b}}\\ \widehat{\mathbf{u}}\end{array}\right]=\left[\begin{array}{c}{\mathbf{X}}^{\prime}{\mathbf{R}}^{-1}\mathbf{y}\\ {\mathbf{W}}^{\prime}{\mathbf{R}}^{-1}\mathbf{y}\end{array}\right],$$

where $${\mathbf{H}}^{-1}={\mathbf{A}}^{-1}+\left[\begin{array}{cc}\boldsymbol{0}& \boldsymbol{0}\\ \boldsymbol{0}& {\mathbf{G}}_{\text{C}}^{-1}-{\mathbf{A}}_{\text{gg}}^{-1}\end{array}\right]$$, ${\mathbf{G}}_{\text{C}}$ is the genomic relationship matrix, $\mathbf{A}$ is the pedigree-based relationship matrix among all animals, and ${\mathbf{A}}_{\text{gg}}$ is the pedigree-based relationship matrix among the genotyped animals. The ${\mathbf{G}}_{\text{C}}$ matrix will be expressed in the following with equal or marker-specific weights.

The MME (3) is for the original single-step GBLUP model [3, 4] that applies the same genomic relationship matrix to all traits and their covariances. This formulation can be used with the same set of marker weights applied to each trait. In the following, we will use the single-step GTBLUP approach but any single-step GBLUP model will follow the same principles [15].

According to the ssGTBLUP model, assume that the genomic relationship matrix has the form ${\mathbf{G}}_{\text{C}}={\mathbf{G}}_{\text{m}}+\mathbf{C}$, where ${\mathbf{G}}_{\text{m}}$ is the genomic and $\mathbf{C}$ the regularization matrix. Two common regularization matrices are: ${\mathbf{C}}_{\text{e}}=e\mathbf{I}$ and ${\mathbf{C}}_{w}=w{\mathbf{A}}_{\text{gg}}$, where $e$ is a small number such as 0.01 and $w$ is the residual polygenic proportion equal to the proportion of genetic variance left unexplained by the markers used. Let ${\mathbf{G}}_{\text{m}}={\mathbf{Z}}_{\text{c}}\mathbf{B}{\mathbf{Z}}_{\text{c}}^{\mathbf{^{\prime}}}$, where ${\mathbf{Z}}_{\text{c}}$ is an $n$ by $m$ matrix of centered marker genotypes, $n$ is the number of genotyped animals, $m$ is the number of SNPs, and $\mathbf{B}$ is an $m$ by $m$ diagonal scaling and weighting matrix. VanRaden [16] method 1 assumes equal weights for each marker, resulting in a scaling matrix $\mathbf{B}$ that is equal to ${\mathbf{B}}_{VR,e}=\mathbf{I}\frac{1}{s}$ for ${\mathbf{C}}_{\text{e}}$ and ${\mathbf{B}}_{VR,w}=\mathbf{I}\frac{1-w}{s}$ for ${\mathbf{C}}_{w}$ where the scaling constant is $s=2{\sum }_{i=1}^{m}{p}_{i}{(1-p}_{i})$. Marker weights can be represented by a diagonal weighting matrix $\mathbf{D}$, e.g., ${\mathbf{B}}_{D,w}=\mathbf{D}\frac{1-w}{s}$, where it is assumed that $\text{tr}(\mathbf{D})=m$, i.e., the average weight is 1. We will assume all weights to be greater than zero. For simplicity, we will omit subscripts for the different regularization matrices, i.e., $e$ or $w$.

The inverse of the genomic relationship matrix in the MME of a single-step GTBLUP can be expressed using the Woodbury identity [7, 17]. When the same SNP weights are used for all traits, the inverse can be expressed as ${\mathbf{G}}_{\text{C}}^{-1}={\mathbf{C}}^{-1}-{\mathbf{C}}^{-1}{\mathbf{Z}}_{c}{\mathbf{K}}^{-1}{\mathbf{Z}}_{c}^{\mathbf{^{\prime}}}$, where $\mathbf{K}={\mathbf{Z}}_{\text{c}}^{\boldsymbol{^{\prime}}}{\mathbf{C}}^{-1}{\mathbf{Z}}_{\text{c}}+{\mathbf{B}}^{-1}$, where the scaling matrix $\mathbf{B}$ has the marker weights. When the traits have different weights, the inverse genomic relationship matrix has different weights, also for the trait-by-trait covariances. In practice, this may lead to a large RAM storage need because the inverses of matrix $\mathbf{K}$ has to be computed with different scaling matrices $\mathbf{B}$ and stored for $T(T+1)/2$ cases.

#### Trait-specific marker weights in single-step SNPBLUP

The MME of the multi-trait single-step SNPBLUP model equivalent to Model (3) with the same marker weights for all traits are:

4 $$\left[\begin{array}{ccc}{\mathbf{X}}^{\mathbf{^{\prime}}}{\mathbf{R}}^{-1}\mathbf{X}& {\mathbf{X}}^{\mathbf{^{\prime}}}{\mathbf{R}}^{-1}\mathbf{W}& \boldsymbol{0}\\ {\mathbf{W}}^{\mathbf{^{\prime}}}{\mathbf{R}}^{-1}\mathbf{X}& {\mathbf{W}}^{\mathbf{^{\prime}}}{\mathbf{R}}^{-1}\mathbf{W}+{\mathbf{G}}_{0}^{-1}\otimes {\mathbf{H}}_{\text{C}}^{-1}& -{\mathbf{G}}_{0}^{-1}\otimes {\mathbf{K}}_{\text{C}}\\ \boldsymbol{0}& -{\mathbf{G}}_{0}^{-1}\otimes {\mathbf{K}}_{\text{C}}^{\boldsymbol{^{\prime}}}& {\mathbf{G}}_{0}^{-1}\otimes \mathbf{K}\end{array}\right]\left[\begin{array}{c}\widehat{\mathbf{b}}\\ \widehat{\mathbf{u}}\\ \widehat{\mathbf{g}}\end{array}\right]=\left[\begin{array}{c}{\mathbf{X}}^{\prime}{\mathbf{R}}^{-1}\mathbf{y}\\ {\mathbf{W}}^{\prime}{\mathbf{R}}^{-1}\mathbf{y}\\ \boldsymbol{0}\end{array}\right],$$

where $${\mathbf{H}}_{\text{C}}^{-1}={\mathbf{A}}^{-1}+\left[\begin{array}{cc}\boldsymbol{0}& \boldsymbol{0}\\ \boldsymbol{0}& {\mathbf{C}}^{-1}-{\mathbf{A}}_{\text{gg}}^{-1}\end{array}\right]$$, matrix $${\mathbf{K}}_{\text{C}}=\left[\begin{array}{c}\boldsymbol{0}\\ {\mathbf{C}}^{-1}{\mathbf{Z}}_{\text{c}}\end{array}\right]$$ is from the estimated marker effects $\widehat{\mathbf{g}}$ to genotypes, and $\mathbf{K}={\mathbf{Z}}_{\text{c}}^{\boldsymbol{^{\prime}}}{\mathbf{C}}^{-1}{\mathbf{Z}}_{\text{c}}+{\mathbf{B}}^{-1}$. As explained in [14], this formulation of the single-step model allows an iterative solver to be programmed such that no genomic related matrices need to be precomputed in order to have an efficient solver, in contrast to using the MME (3). Instead, all computations can be performed on-the-fly. The scaling matrix ${\mathbf{B}}_{D}$, which includes the marker weights, can be used in the computations. However, in MME (4), the same marker weights are assumed for each trait. Furthermore, note that the covariance matrix for the *k*-th marker is ${\mathbf{G}}_{0}{\mathbf{B}}_{D,k}$ where ${\mathbf{B}}_{D,k}$ is the *k*-th diagonal element of ${\mathbf{B}}_{D}$. Thus, weights modify the variances of the marker effects.

Trait-specific marker weights require some changes to MME (4). Following Liu et al. [11], the covariance matrix for the effects of the $k$-th marker is:

5 $${\mathbf{V}}_{\text{g},k}=\left[\begin{array}{cccc}{\mathbf{g}}_{\text{0,11}}{\mathbf{B}}_{11,k}& {\mathbf{g}}_{\text{0,12}}{\mathbf{B}}_{12,k}& \dots & {\mathbf{g}}_{\text{0,1}T}{\mathbf{B}}_{1T,k}\\ {\mathbf{g}}_{\text{0,21}}{\mathbf{B}}_{21,k}& {\mathbf{g}}_{\text{0,22}}{\mathbf{B}}_{22,k}& \dots & {\mathbf{g}}_{0,2T}{\mathbf{B}}_{1T,k}\\ \vdots & \vdots & \ddots & \vdots \\ {\mathbf{g}}_{0,T1}{\mathbf{B}}_{\text{T}1,k}& {\mathbf{g}}_{0,T2}{\mathbf{B}}_{T2,k}& \dots & {\mathbf{g}}_{0,TT}{\mathbf{B}}_{TT,k}\end{array}\right],$$

where ${\mathbf{g}}_{0,ij}$ is the genetic covariance between traits *i* and *j* in ${\mathbf{G}}_{0}$ and ${\mathbf{B}}_{ij,k}$ is the weight for marker $k$ in the position of traits $i$ and $j$. Every matrix ${\mathbf{V}}_{\text{g},k}$ is required to be positive definite. Because the marker effects are assumed to be independent, the marker effect covariance matrix is block diagonal, with blocks of ${\mathbf{V}}_{\text{g},k}$. This requirement can be relaxed without changing Eq. (7) below. Matrix ${\mathbf{V}}_{\text{g},k}$ is a $T$ by $T$ matrix with marker (co)variances. In other words, the use of weights is equivalent to assuming marker-specific genetic (co)variance matrices. In the original notation of the marker effect vector $\mathbf{g}$, the marker (co)variance matrix for all markers can be written as:

6 $${\mathbf{V}}_{\text{g}}=\left[\begin{array}{cccc}{\mathbf{g}}_{\text{0,11}}{\mathbf{B}}_{11}& {\mathbf{g}}_{\text{0,12}}{\mathbf{B}}_{12}& \dots & {\mathbf{g}}_{\text{0,1}T}{\mathbf{B}}_{1T}\\ {\mathbf{g}}_{\text{0,21}}{\mathbf{B}}_{21}& {\mathbf{g}}_{\text{0,22}}{\mathbf{B}}_{22}& \dots & {\mathbf{g}}_{0,2T}{\mathbf{B}}_{1T}\\ \vdots & \vdots & \ddots & \vdots \\ {\mathbf{g}}_{0,T1}{\mathbf{B}}_{\text{T}1}& {\mathbf{g}}_{0,T2}{\mathbf{B}}_{T2}& \dots & {\mathbf{g}}_{0,TT}{\mathbf{B}}_{TT}\end{array}\right].$$

This formulation allows for different marker weight matrices within each trait as well as between traits, which are in the diagonal matrices ${\mathbf{B}}_{ij}={\mathbf{D}}_{ij}\frac{1}{s}$ for trait $i$ and $j$ with marker weight matrix ${\mathbf{D}}_{ij}$. Note that the average weight in ${\mathbf{D}}_{ij}$ is 1. When the same weights are used for all traits, Eq. (6) can be simply expressed as ${\mathbf{G}}_{0}\otimes \mathbf{B}$.

The marker weight matrix ${\mathbf{D}}_{ij}$ may be unknown for some combinations of traits $i$ and $j$. When no marker weights are used, $\mathbf{D}$ is an identity matrix. However, when the marker weights are known only for the traits, i.e., ${\mathbf{D}}_{ii}$ for all *i* is known, the “covariance” weight matrix can be set to have a “correlation” of one between traits. Thus, ${\mathbf{D}}_{ij}={\left({\mathbf{D}}_{ii}{\mathbf{D}}_{jj}\right)}^{0.5}$. This will lead to a covariance structure that is equal to ${{\mathbf{V}}_{\text{g},k}={\mathbf{D}}_{(k)}^{0.5}\mathbf{G}}_{0}{\mathbf{D}}_{(k)}^{0.5}$, where the diagonal matrix ${\mathbf{D}}_{(k)}$ has the weights for all traits of marker *k*.

In single-step multi-trait SNPBLUP, the covariance matrix for the breeding values and the SNP effects is:

7 $$\text{Var}\left(\begin{array}{c}{\mathbf{u}}_{n}\\ {\mathbf{u}}_{\text{g}}\\ \mathbf{g}\end{array}\right)=\left[\begin{array}{ccc}\left({\mathbf{I}}_{T}\otimes \mathbf{T}\right){\mathbf{G}}_{\text{gg}}\left({\mathbf{I}}_{T}\otimes \mathbf{T}\boldsymbol{^{\prime}}\right)+{\mathbf{G}}_{0}\otimes {\left({\mathbf{A}}^{nn}\right)}^{-1}& \left({\mathbf{I}}_{T}\otimes \mathbf{T}\right){\mathbf{G}}_{\text{gg}}& \left({\mathbf{I}}_{T}\otimes \mathbf{T}{\mathbf{Z}}_{\text{c}}\right){\mathbf{V}}_{\text{g}}\\ {\mathbf{G}}_{\text{gg}}\left({\mathbf{I}}_{T}\otimes \mathbf{T}\boldsymbol{^{\prime}}\right)& {\mathbf{G}}_{\text{gg}}& \left({\mathbf{I}}_{T}\otimes {\mathbf{Z}}_{\text{c}}\right){\mathbf{V}}_{\text{g}}\\ {\mathbf{V}}_{\text{g}}\left({\mathbf{I}}_{T}\otimes {\left(\mathbf{T}{\mathbf{Z}}_{\text{c}}\right)}^{\boldsymbol{^{\prime}}}\right)& {\mathbf{V}}_{\text{g}}\left({\mathbf{I}}_{T}\otimes {\mathbf{Z}}_{\text{c}}^{\boldsymbol{^{\prime}}}\right)& {\mathbf{V}}_{\text{g}}\end{array}\right],$$

where the genetic variance for the breeding values of the genotyped animals is $\text{Var}\left({\mathbf{u}}_{g}\right)={\mathbf{G}}_{\text{gg}}=\left({\mathbf{I}}_{T}\otimes {\mathbf{Z}}_{\text{c}}\right){\mathbf{V}}_{\text{g}}\left({\mathbf{I}}_{T}\otimes {\mathbf{Z}}_{\text{c}}^{\boldsymbol{^{\prime}}}\right)+{\mathbf{G}}_{0}\otimes \mathbf{C}$, where ${\mathbf{V}}_{\text{g}}$ is the marker covariance matrix, ${\mathbf{A}}^{nn}$ is the submatrix for the non-genotyped individuals in the ${\mathbf{A}}^{-1}$ matrix, and $\mathbf{T}={\mathbf{A}}_{n\text{g}}{\mathbf{A}}_{\text{gg}}^{-1}$ with ${\mathbf{A}}_{n\text{g}}$ representing the relationship matrix between the non-genotyped and the genotyped individuals. Note that Eq. (7) is a generalization of equation A15 in [11]. In particular, when all traits and markers have the same weights, i.e. ${\mathbf{V}}_{\text{g}}={\mathbf{G}}_{0}\otimes \mathbf{B}$, the upper left corner of Eq. (7) can be written ${\mathbf{G}}_{0}\otimes \left({\mathbf{A}}_{nn}+{\mathbf{A}}_{n\text{g}}{\mathbf{A}}_{\text{gg}}^{-1}\left({{\mathbf{G}}_{C}-\mathbf{A}}_{\text{gg}}\right){\mathbf{A}}_{\text{gg}}^{-1}{\mathbf{A}}_{\text{g}n}\right)$, which is a multi-trait form of the single-step SNPBLUP model variance of ${\mathbf{u}}_{n}$ in [14].

The genetic covariance matrix ${\mathbf{G}}_{0}$ is included in the covariance matrix ${\mathbf{V}}_{\text{g}}$ (7). Thus, it is no longer possible to separate the relationship matrix and the genetic covariance matrix. In the usual notation as in [11], we denote variance matrix (7) as the ${\mathbf{H}}_{\text{g}}$ matrix. It can be shown that its inverse needed in the MME can be expressed in such a way that it can be used in solving the MME with only small modifications in the standard solving approach. When ${\mathbf{C}}_{w}=w{\mathbf{A}}_{\text{gg}}$, the inverse of ${\mathbf{H}}_{\text{g}}$ is:

$${\mathbf{H}}_{\text{g}}^{-1}=\left[\begin{array}{cc}{\mathbf{G}}_{0}^{-1}\otimes \left[\begin{array}{cc}{\mathbf{A}}^{11}& {\mathbf{A}}^{12}\\ {\mathbf{A}}^{21}& {\mathbf{A}}^{22}+\left(\frac{1}{w}-1\right){\mathbf{A}}_{\text{gg}}^{-1}\end{array}\right]& {-\mathbf{G}}_{0}^{-1}\otimes \left[\begin{array}{c}\boldsymbol{0}\\ \frac{1}{w}{\mathbf{A}}_{\text{gg}}^{-1}{\mathbf{Z}}_{\text{c}}\end{array}\right]\\ {-\mathbf{G}}_{0}^{-1}\otimes \left[\begin{array}{cc}\boldsymbol{0}& \frac{1}{w}{{\mathbf{Z}}_{\text{c}}^{\boldsymbol{^{\prime}}}\mathbf{A}}_{\text{gg}}^{-1}\end{array}\right]& {\mathbf{G}}_{0}^{-1}\otimes \left(\frac{1}{w}{\mathbf{Z}}_{\text{c}}^{\boldsymbol{^{\prime}}}{\mathbf{A}}_{\text{gg}}^{-1}{\mathbf{Z}}_{\text{c}}\right)+{\mathbf{V}}_{\text{g}}^{-1}\end{array}\right].$$

This inverse is similar to the matrix with equal marker weights. The only difference is the need for ${\mathbf{V}}_{\text{g}}^{-1}$ instead of ${\mathbf{G}}_{0}^{-1}\otimes {\mathbf{B}}^{-1}$ in the model with equal marker weights for all traits. Note that this inverse can be derived similarly for regularization matrix ${\mathbf{C}}_{e}=e\mathbf{I}$.

#### Mixed model equations with trait-specific marker weights

The breeding value of a genotyped animal is expressed as ${\mathbf{u}}_{\text{g}}=\left({\mathbf{I}}_{L}\otimes {\mathbf{Z}}_{\text{c}}\right)\mathbf{g}+{\mathbf{a}}_{\text{g}},$ where ${\mathbf{a}}_{\text{g}}$ is the term due to the regularization [14]. In the previous models corresponding to MME (3) and (4), $\text{Var}\left({\mathbf{u}}_{\text{g}}\right)={\mathbf{G}}_{0}\otimes \left({\mathbf{Z}}_{\text{c}}\mathbf{B}{\mathbf{Z}}_{\text{c}}^{\boldsymbol{^{\prime}}}+\mathbf{C}\right)$. This allows the use of the same genomic relationship matrix in the MME for all traits. In order to use trait-specific marker weights, MME (3) would require a separate genomic relationship matrix for every trait and for every trait-by-trait covariance. In the MME of the single-step SNPBLUP (4), this only requires the lower right-hand matrix of the coefficient matrix to be changed by using the ${\mathbf{V}}_{\text{g}}$ matrix. The MME of trait-specific single-step SNPBLUP then are:

8 $$\left[\begin{array}{ccc}{\mathbf{X}}^{\mathbf{^{\prime}}}{\mathbf{R}}^{-1}\mathbf{X}& {\mathbf{X}}^{\mathbf{^{\prime}}}{\mathbf{R}}^{-1}\mathbf{W}& \boldsymbol{0}\\ {\mathbf{W}}^{\mathbf{^{\prime}}}{\mathbf{R}}^{-1}\mathbf{X}& {\mathbf{W}}^{\mathbf{^{\prime}}}{\mathbf{R}}^{-1}\mathbf{W}+{\mathbf{G}}_{0}^{-1}\otimes {\mathbf{H}}_{\text{C}}^{-1}& -{\mathbf{G}}_{0}^{-1}\otimes {\mathbf{K}}_{\text{C}}\\ \boldsymbol{0}& -{\mathbf{G}}_{0}^{-1}\otimes {\mathbf{K}}_{\text{C}}^{\boldsymbol{^{\prime}}}& {\mathbf{G}}_{0}^{-1}\otimes {\mathbf{Z}}_{\text{c}}^{\boldsymbol{^{\prime}}}{\mathbf{C}}^{-1}{\mathbf{Z}}_{\text{c}}+{\mathbf{V}}_{\text{g}}^{-1}\end{array}\right]\left[\begin{array}{c}\widehat{\mathbf{b}}\\ \widehat{\mathbf{u}}\\ \widehat{\mathbf{g}}\end{array}\right]=\left[\begin{array}{c}{\mathbf{X}}^{\prime}{\mathbf{R}}^{-1}\mathbf{y}\\ {\mathbf{W}}^{\prime}{\mathbf{R}}^{-1}\mathbf{y}\\ 0\end{array}\right].$$

Matrix ${\mathbf{V}}_{\text{g}}^{-1}$ can be easily computed because it is very sparse. According to Eqs. (5) and (6) of the ${\mathbf{V}}_{\text{g}}$ matrix, every marker has a matrix of size equal to the number of traits, where the marker weights for each trait (and their covariances) are multiplied by an appropriate genetic (co)variance. Liu et al. [11] expressed this submatrix between traits for a marker using the genetic standard deviations and genetic correlations, with a weight equal to one for the markers. Note that they expressed the submatrix for marker $i$ in ${\mathbf{V}}_{\text{g}}$ by ${\mathbf{B}}_{ii}$. Thus, when there are six traits and 50,000 markers, inverses of 6 by 6 matrices need to be computed 50,000 times. The added computation due to making ${\mathbf{V}}_{\text{g}}^{-1}$ is relatively small in comparison to a model without marker weights when the number of genotyped individuals is large.

### Data and models

#### Simulation

The single-step genomic models with and without trait-specific marker weights were tested using simulated data. AlphaSimR [18] was used to simulate 10 generations after creating the base population. In general, the simulation of a single population was like in [19] for a single population with production and adaptation traits selected with equal weight, except for some differences due to the use of different simulation software.

The cattle species history was used for generating the founder population haplotypes, as programmed in AlphaSimR, with an effective population size of 280. After the historical simulation, a base population of 2800 males and 2800 females was generated. The base population individuals were mated randomly, each mating producing one offspring. After the base population, a selection index was used to select the best 200 males and 2800 females to form the breeding population. The selection index used own phenotypes for each trait, weighing them equally. Each mating in the breeding population produced one offspring, either male or female, at equal numbers. In every subsequent generation, the best 200 males and 2800 females were selected among the group consisting of the current breeding population and the offspring produced by the random mating of the breeding animals. After 10 generations of selection and breeding, the final pedigree consisted of 16,940 females and 16,940 males.

Every simulated individual had phenotypes for correlated production and adaptation traits, with heritabilities of 0.3 and 0.1, respectively, a genetic correlation of − 0.3, and a residual correlation of zero. The genome consisted of 30 chromosomes. The two traits were pleiotropic and determined by 900 QTL, i.e., 30 QTL per chromosome, which were randomly sampled from the simulated segregating sites, utilizing cattle demographic history to ensure consistent spacing across both individual chromosomes and the entire genome. The QTL effects for the two traits were simulated from a Gamma density with shape 0.4 and scale 1.0. The simulation was replicated ten times.

#### Data for single-step evaluations

Data used for the single-step genomic evaluations was extracted from the simulated data. Only phenotypes from the females were kept, excluding those from the last generation. The pedigree included 33,880 animals, of which 15,540 had phenotypes. Genotypes on 54,000 SNPs were used from the sires of females and from all animals in the last three generations. There were about 11,726 animals with genotypes, of which 2800 were validation animals in the last generation, 5600 were from the two generations before the last generation, and the rest were sires of females before these generations. The basic genomic data consisted of 54,000 SNPs. Two additional genomic datasets were formed that describe a situation where genome-wide association studies have located QTL. In these cases, genotypes of the largest QTL that, together, explained either 5 or 20% of the total genetic variance were included, resulting in, respectively, 54,010 and 54,048 markers included in the analyses.

#### Single-step models and comparison statistics

Breeding values were estimated using single-step genomic models, in which the markers had either no weights (i.e. equal weights across SNPs and traits), common weights across the traits, or trait-specific weights. For the marker-weighted models, the weights were computed using single-trait BayesA, as posterior means of the marker-specific scaling values [20].

For each replicate, we conducted six separate BayesA analyses, one for each trait and genomic data combination, resulting in 60 BayesA analyses. The phenotypes of the BayesA analyses were deregressed breeding values, which were derived from the pedigree-based multi-trait animal model EBV. The deregression computations used values of only those individuals who had either own or progeny phenotypes. Weights used in the deregression calculations were computed from the animal model by the reverse reliability estimation approach described in [21]. The deregressed values and their weights were used in the BayesA analyses to compute the weights to be used for the marker-weighted single-step models. These marker weights from BayesA were standardized to have a mean of 1.

Three models with the common marker weights were used. In two models, either the production or the adaptation trait weights were used. In the third case, the average of the standardized production and adaptation weights was used. The trait-specific marker-weighted model used the production and adaptation weights for the respective traits. In all trait-specific marker-weighted models, the correlation of weights between the traits was equal to 1.

In all analyses that used genotype data, the genotype matrix was centered and scaled using base population allele frequencies estimated using the Bpop software [22] and a scaling constant $s=2{\sum }_{i=1}^{m}{p}_{i}{(1-p}_{i})$ that was also based on the estimated base population allele frequencies. The residual polygenic proportion was set to 5%. The ssSNPBLUP models used the full pedigree and the true variance components used for simulation. The GEBV obtained from the single-step models were used to compute following validation statistics for the genotyped individuals in the last generation: the correlation between the true BV and GEBV and linear regression of the true BV on GEBV.

#### Solving the single-step models

All single-step models were solved with the software MiX99 [23, 24], which uses the preconditioned conjugate gradient (PCG) and iteration on data methods. A block diagonal preconditioner was used, where the blocks were of the size equal to the number of traits, the blocks were within each level of an effect, and their element values were as in the coefficient matrix of the MME. However, for marker effect *k*, the block was ${\mathbf{G}}_{0}^{-1}{\mathbf{c}}_{k}+{\mathbf{V}}_{\text{g},k}^{-1}$ where ${\mathbf{c}}_{k}=\frac{1}{w}{{{\mathbf{Z}}_{\text{c},k}^{\mathbf{^{\prime}}}\mathbf{D}}_{\text{A}}\mathbf{Z}}_{\text{c},k}$, ${\mathbf{Z}}_{\text{c},k}$ is *k*-th column in the ${\mathbf{Z}}_{\text{c}}$ matrix and ${\mathbf{D}}_{\text{A}}$ is the diagonal of matrix ${\mathbf{A}}_{\text{gg}}^{-1}$. The convergence criterion for the PCG iteration was the relative difference between the left- and right-hand sides of the MME:

$${\text{C}}_{\text{r}}=\sqrt{\frac{{\left({\mathbf{C}}_{\text{MME}}{\mathbf{s}}^{[k]}-{\mathbf{r}}_{\text{MME}}\right)}^{\mathbf{^{\prime}}}\left({\mathbf{C}}_{\text{MME}}{\mathbf{s}}^{[k]}-{\mathbf{r}}_{\text{MME}}\right)}{{\mathbf{r}}_{\text{MME}}\mathbf{^{\prime}}{\mathbf{r}}_{\text{MME}}}},$$

where ${\mathbf{C}}_{\text{MME}}$ is the coefficient matrix of MME, ${\mathbf{s}}^{[k]}$ is the vector of solutions at round $k$, and ${\mathbf{r}}_{\text{MME}}$ is the right-hand side vector. For all evaluations, convergence was assumed to be reached when ${\text{C}}_{\text{r}}$ < 10^–7^. We report the average computing time per iteration and the average number of PCG iterations across the 10 replicates, as well as the average of the standard deviation of the estimated marker weights across the replicates which measures the deviation in the MME with equal or different marker weights.

## Results

The three marker datasets required about the same number of iterations to converge and about the same computing time per iteration when no weights were used in the single-step model (Table 1). Likewise, the regression and prediction accuracy values were very similar for the three marker data sets, even when QTL were included among the SNP markers. In other words, there was no increase in the prediction accuracy even when the marker set contained QTL that together explained 20% of the genetic variation.

**Table 1:** Regression of the true breeding value on the estimated breeding value (b) for production and adaptation, and their correlation (r) in the last generation (standard error in the parenthesis), for the single-step models without marker weights using SNP data containing QTL that together explained 0, 5, or 20% of the genetic variance

p QTL	N	Time	Production		Adaptation	
			b	r	b	r
0	421	0.46	1.01 (0.010)	0.68 (0.006)	0.97 (0.021)	0.52 (0.010)
5	426	0.46	1.01 (0.010)	0.68 (0.006)	0.97 (0.021)	0.53 (0.010)
20	437	0.46	1.02 (0.010)	0.68 (0.006)	0.98 (0.021)	0.53 (0.010)

p QTL = QTL that explained p% of the genetic variance included in the model as SNPs in addition to the 54,000 SNPs

N = Average number of iterations until convergence

Time = Average PCG computing time per iteration in seconds

The use of common marker weights for both traits increased the number of iterations until convergence considerably (Table 2), often about 3 to 4 times that of the model without marker weights. The increase tended to be larger the more the marker weights deviated from 1, which was indicated by a larger average standard deviation of the weights. The standard deviation of the marker weights computed for production tended to be higher than those for adaptation, and this difference was represented in a greater number of PCG iterations. Including marker weights did not affect computing times per iteration.

**Table 2:** Regression of the true breeding value on the estimated breeding value (b) for production and adaptation, and their correlation (r) in the last generation (standard error in the parenthesis), for the single-step models with common marker weights using SNP data containing QTL that together explained 0, 5, or 20% of the genetic variance

Weights	p QTL	N	Time	SD	Production		Adaptation	
					b	r	b	r
P	0	1760	0.45	6.80	0.95 (0.011)	0.74 (0.008)	0.97 (0.022)	0.53 (0.009)
A	0	1459	0.45	2.37	1.01 (0.010)	0.68 (0.006)	0.93 (0.018)	0.56 (0.010)
AP	0	1706	0.45	5.24	0.95 (0.011)	0.73 (0.008)	0.96 (0.020)	0.55 (0.010)
P	5	1741	0.46	8.74	0.94 (0.011)	0.75 (0.009)	0.99 (0.021)	0.53 (0.009)
A	5	1589	0.46	3.36	1.02 (0.010)	0.68 (0.006)	0.93 (0.018)	0.58 (0.011)
AP	5	1743	0.46	6.73	0.94 (0.012)	0.75 (0.009)	0.98 (0.018)	0.57 (0.010)
P	20	1836	0.46	8.82	0.93 (0.009)	0.78 (0.008)	0.99 (0.023)	0.54 (0.009)
A	20	1610	0.46	3.72	1.02 (0.010)	0.68 (0.006)	0.93 (0.017)	0.59 (0.010)
AP	20	1808	0.46	6.83	0.94 (0.010)	0.78 (0.008)	0.98 (0.018)	0.58 (0.010)

The used marker weights had been computed for production (P) and adaptation (A), or was their average (AP)

p QTL = QTL that explained p% of the genetic variance included in the model as SNPs in addition to the 54,000 SNPs

N = Average number of iterations until convergence

Time = Average PCG computing time per iteration in seconds

SD = Average standard deviation of marker weights

Including common weights for the two traits improved the prediction accuracy of the trait whose weights were used, but not necessarily the other trait. Prediction accuracy increased only slightly when more QTLs were included in the marker set. When the marker weights were based on the average estimated weights across the two traits, prediction accuracy increased for both traits but did not always reach the prediction accuracy achieved when using the weights estimated for the matching trait, but the difference was quite small. As prediction accuracy increased, the regression coefficients reduced further below one, in particular for production, suggesting an increase in bias.

The use of marker-specific weights in the single-step SNPBLUP model (Table 3) took more iterations to converge than the models without weights but a similar number as models with equal weights for both traits. The average number of iterations required was not as high as in models that used the marker weights estimated for production, but not as low as those required for models that used weights estimated for adaptation. As with the model with equal marker weights for both traits, the larger the average standard deviation of the marker weights the greater the number of iterations until convergence. The standard deviations for the traits are shown in Table 2. For example, the average standard deviations for the first model in Table 3 are 6.80 and 2.37 for production and adaptation, respectively, from the first two models in Table 2. As more QTL were included among the SNP markers, the average standard deviations increased, thus increasing the number of iterations until convergence (Table 3). Computing times per iteration were about the same.

**Table 3:** Regression of the true breeding value on the estimated breeding value (b) for production and adaptation, and their correlation (r) in the last generation (standard error in parenthesis) for single-step models with trait-specific marker weights using SNP data containing QTL that together explained 0, 5, or 20% of the genetic variance

p QTL	N	Time	Production		Adaptation	
			b	r	b	r
0	1557	0.45	0.95 (0.011)	0.74 (0.008)	0.93 (0.018)	0.56 (0.010)
5	1651	0.46	0.94 (0.011)	0.75 (0.009)	0.93 (0.017)	0.57 (0.011)
20	1735	0.46	0.93 (0.009)	0.78 (0.008)	0.93 (0.017)	0.59 (0.010)

p QTL = QTL that explained p% of the genetic variance included in the model as SNPs in addition to the 54,000 SNPs

N = Average number of iterations until convergence

Time = Average PCG computing time per iteration in seconds

Prediction accuracies for the trait-specific marker weight models were approximately the same as those of models with common weights when the weights were based on their own trait. However, the regression coefficients were slightly further below 1, suggesting an increase in bias. In addition, the regression coefficients of the trait-specific marker weight models were always lower than those without weights in Table 1, suggesting an increase in bias in exchange for an increase in the prediction accuracy.

## Discussion

The use of weights in single-step SNPBLUP increased prediction accuracies but also extended computing time due to the need for more iterations until convergence. The increase in prediction accuracy was substantial for the production trait (an increase up to 0.10) but smaller for the adaptation trait (an increase up to 0.06). Analyzing large real data is likely to yield significantly smaller improvements compared to the gains observed with this small simulated dataset.

The simple single-trait approach to compute the weights (BayesA) and the assumption of one for the correlation between the trait weights may have limited the accuracy increase for adaptation. Accurate estimation of the weights requires more investigation and larger genotype data set. Alternatively, a multi-trait Bayesian analysis can be used to estimate marker-specific variance–covariance matrices [13]. A multi-trait model can enhance the prediction accuracy by more correctly accounting for the correlations between traits for genome regions that affect one trait more than the other. For example, in our study, all correlations between traits within markers were negative, driven by the overall genetic covariance matrix ${\mathbf{G}}_{0}$. Thus, although the use of marker weights can increase prediction accuracy, a better approach than used here may be available for a multi-trait single-step model. Because the estimation of marker weights by a multi-trait Bayesian model can be time consuming, the same weights for the traits can be used in successive single-step SNPBLUP evaluations as data accumulates, but with a possible decrease in prediction accuracy.

We assumed that all weights were non-zero to make the marker covariance matrix ${\mathbf{V}}_{\text{g}}$ invertible. Some markers may have a zero weight when the weights have been estimated with a multi-trait model [13] or a variable selection model like BayesB [20]. We avoided this problem by using single-trait BayesA and setting the correlation between traits in the marker weight matrix equal to one. Marker weights estimated by another approach can be used after scaling them to be one on average. However, any zero weights must be replaced by a small positive value, e.g. 10^–6^. Because the ${\mathbf{V}}_{\text{g}}$ matrix has to be positive definite, simply replacing zero weights by a small positive value may not be enough.

We presented a single-step SNPBLUP model with trait-specific marker weights that required only a small change in the MME of a regular single-step SNPBLUP. The change involved computation of the marker variance matrix ${\mathbf{V}}_{\text{g}}$ and its inverse, which is feasible even for large numbers of genotyped individuals because the values in the ${\mathbf{V}}_{\text{g}}$ matrix are not affected by the genotype data and the ${\mathbf{V}}_{\text{g}}$ matrix has a block-diagonal structure, allowing for easy computation and low RAM use. However, this did lead to poor convergence and a several-fold increase in the solving time. In practice, we have observed that the convergence is poorer when the weights deviate more from one, i.e., from the standard single-step model without weights. The commonly used single-step GBLUP and GTBLUP approaches tend to have better convergence properties [14] but have different kinds of computational challenges when including trait-specific marker weights because of the need to compute and store matrices for every trait and for every trait-by-trait combination of marker weights.

The regular single-step GBLUP approach with trait-specific marker weights will be computationally demanding. Consider a two-trait model. When the markers have different weights by trait, the genetic covariance matrix of the genotyped individuals can be expressed as:

$${\mathbf{G}}_{u}=\text{Var}\left(\begin{array}{c}{\mathbf{u}}_{1,\text{g}}\\ {\mathbf{u}}_{2,\text{g}}\end{array}\right)=\left[\begin{array}{cc}{\mathbf{g}}_{\text{0,11}}\left({\mathbf{Z}}_{\text{c}}{\mathbf{B}}_{11}{\mathbf{Z}}_{\text{c}}^{\boldsymbol{^{\prime}}}+\mathbf{C}\right)& {\mathbf{g}}_{\text{0,12}}\left({\mathbf{Z}}_{\text{c}}{\mathbf{B}}_{12}{\mathbf{Z}}_{\text{c}}^{\boldsymbol{^{\prime}}}+\mathbf{C}\right)\\ {\mathbf{g}}_{\text{0,21}}\left({\mathbf{Z}}_{\text{c}}{\mathbf{B}}_{21}{\mathbf{Z}}_{\text{c}}^{\boldsymbol{^{\prime}}}+\mathbf{C}\right)& {\mathbf{g}}_{\text{0,22}}\left({\mathbf{Z}}_{\text{c}}{\mathbf{B}}_{22}{\mathbf{Z}}_{\text{c}}^{\boldsymbol{^{\prime}}}+\mathbf{C}\right)\end{array}\right]=\left({\mathbf{I}}_{2}\otimes {\mathbf{Z}}_{\text{c}}\right)\left[\begin{array}{cc}{\mathbf{g}}_{\text{0,11}}{\mathbf{B}}_{11}& {\mathbf{g}}_{\text{0,12}}{\mathbf{B}}_{12}\\ {\mathbf{g}}_{\text{0,21}}{\mathbf{B}}_{21}& {\mathbf{g}}_{\text{0,22}}{\mathbf{B}}_{22}\end{array}\right]\left({\mathbf{I}}_{2}\otimes {\mathbf{Z}}_{\text{c}}^{\boldsymbol{^{\prime}}}\right)+{\mathbf{G}}_{0}\otimes \mathbf{C}=\left({\mathbf{I}}_{2}\otimes {\mathbf{Z}}_{\text{c}}\right){\mathbf{V}}_{\text{g}}\left({\mathbf{I}}_{2}\otimes {\mathbf{Z}}_{\text{c}}^{\boldsymbol{^{\prime}}}\right)+{\mathbf{G}}_{0}\otimes \mathbf{C},$$

where the diagonal matrix ${\mathbf{B}}_{ij}$ has marker weights for traits $i$ and $j$, and ${\mathbf{V}}_{\text{g}}$ is the covariance between the markers. This is no longer in the form of ${\mathbf{G}}_{0}\otimes \left({\mathbf{Z}}_{\text{c}}\mathbf{B}{\mathbf{Z}}_{\text{c}}^{\boldsymbol{^{\prime}}}+\mathbf{C}\right)$, where the Kronecker product allows separate inversion of the genetic covariance and the genomic relationship matrices. Consequently, for $T$ traits, $T(T+1)/2$ genomic relationship matrices must be built and matrix ${\mathbf{G}}_{u}$ of size $Tn$ must be inverted for its use in MME, where $n$ is the number of genotyped individuals. Thus, when the number of genotyped individuals is large, the regular single-step GBLUP will become infeasible due to long computing time and memory needs.

The component-wise ssGTBLUP approach is more efficient than the regular ssGBLUP when no weights are used [7]. However, the use of component-wise ssGTBLUP can become infeasible in practice as well when trait-specific marker weights are used. The Woodbury matrix identity for a model with trait-specific marker weights can be used in the computations to invert ${\mathbf{G}}_{u}$: ${\mathbf{G}}_{u}^{-1}=\left({\mathbf{G}}_{0}^{-1}\otimes {\mathbf{C}}^{-1}\right)-\left({\mathbf{G}}_{0}^{-1}\otimes {\mathbf{C}}^{-1}\right)\left({\mathbf{I}}_{2}\otimes {\mathbf{Z}}_{\text{c}}\right){\left(\left({\mathbf{I}}_{2}\otimes {\mathbf{Z}}_{\text{c}}^{\boldsymbol{^{\prime}}}{\mathbf{C}}^{-1}{\mathbf{Z}}_{\text{c}}\right)+{\mathbf{V}}_{\text{g}}^{-1}\right)}^{-1}\left({\mathbf{I}}_{2}\otimes {\mathbf{Z}}_{\text{c}}^{\boldsymbol{^{\prime}}}\right)\left({\mathbf{G}}_{0}^{-1}\otimes {\mathbf{C}}^{-1}\right)$. In Vandenplas et al. [14], precomputing the components for the regular component-wise ssGTBLUP with 2.61 million genotyped individuals with $m$ = 47,006 SNPs took 22.3 h. The major computational task is due to ${\mathbf{Z}}_{\text{c}}^{\boldsymbol{^{\prime}}}{\mathbf{C}}^{-1}{\mathbf{Z}}_{\text{c}}$. In the model with trait-specific marker weights, making the Kronecker product ${\mathbf{I}}_{2}\otimes {\mathbf{Z}}_{\text{c}}^{\boldsymbol{^{\prime}}}{\mathbf{C}}^{-1}{\mathbf{Z}}_{\text{c}}$ and adding ${\mathbf{V}}_{\text{g}}^{-1}$ to this is computationally not very demanding. However, increasing the number of traits will require a matrix of size $Tm$ to be inverted instead of an $m$ size matrix. When there are 10 traits and 50,000 markers in the model, this matrix would have a size of 500,000 and would take about 1 TB memory when stored as double precision floating point numbers. Thus, while computational challenges with ssGTBLUP do not change as dramatically with an increase in the number of traits compared to ssGBLUP by the use of marker weights, the memory required with the component-wise ssGTBLUP, as in ssGBLUP, can become a bottleneck in a multi-trait model with trait-specific marker weights.

The single-step SNPBLUP approach presented here assumes that the weights have been estimated using a model similar to the single-step SNPBLUP model using the marker weights. In other words, it requires a weight for every marker-trait combination that is included in the model. This is typical in most genetic evaluations, even if they include metafounders [25] or J factors [26]. However, some models are quite complex, such as the reduced rank random regression test-day model for milk production used in the joint Nordic evaluation [27]. For such a model, different weights can be assigned to different random regression covariables, which can differ by trait. Perhaps, the same weights as estimated using 305-day observations for a trait can be used for the covariables within a trait in a random regression model. However, in a reduced rank model, weights must be assigned to functions of trait covariables that are used over traits. Further work is needed to investigate the use of weights estimated using a model other than the marker weighted single-step SNPBLUP model used, and to find computationally efficient methods for estimating marker weights.

## Conclusions

A multi-trait single-step model with individual marker weighting is simple when the weights are the same across traits. Trait-specific marker weights in a multi-trait model can be included in a single-step SNPBLUP model when the weights have been precomputed. This required only small modifications to software for a model without weights. Alternative approaches to single-step SNPBLUP exist but can be computationally more challenging. Weighting increased computational requirements of the iterative solver only slightly. The two-step approach of first estimating the marker weights by trait and then using these in single-step SNPBLUP may provide a computationally feasible strategy to increase prediction accuracy even for large genomic single-step evaluations. However, the number of iterations until convergence increased considerably, suggesting the weights used were not optimal. Convergence became poorer as the estimated marker weights deviated from equal weights. Furthermore, the presence of QTL among the markers used in the genetic evaluation increased prediction accuracy marginally. Thus, further investigation is needed to find a better approach for estimating trait-specific marker weights for a single-step model.

## Data Availability

The code for simulating data with AlphaSimR can be requested from the authors.
